# A Silent Alarm at Occupational Evaluation Two Months after a Normal Painful ECG: A Case of Wellens' syndrome

**DOI:** 10.1155/2015/819205

**Published:** 2015-04-14

**Authors:** Giuseppe Di Stolfo, Sandra Mastroianno, Giovanni De Luca, Domenico Rosario Potenza, Nicola Marchese, Carlo Vigna, Raffaele Fanelli

**Affiliations:** Department of Cardiology, Casa Sollievo della Sofferenza Hospital, San Giovanni Rondo, Foggia, Italy

## Abstract

We describe a case of a 42-year-old man, with a previous episode of angina and a normal ECG and serum cardiac markers, and a two months later finding of biphasic T wave in leads V2-V3 and deeply inverted T wave in V4-V5 at a asymptomatic occupational evaluation. This is a typical ECG pattern of Wellens' syndrome. A subsequent coronary angiography showed a critical stenosis of proximal left anterior descendent. We underline the careful value of prolonged observation in chest pain unit and repetitive ECG evaluation also during pain-free period after an angina episode, to exclude an earlier T wave pseudonormalization.

## 1. Introduction

Chest pain represents one of the principal causes of emergency department access in the Western World. At same time differential diagnosis and right patient management are hampered by overcrowded hospital and overstressed staff. Despite several and effective available tools represented by clinical evaluation, patient history, cardiovascular risk profile, ECG, and serum cardiac markers, a nonnegligible percentage of patients with or at very high risk of coronary syndrome are discharged improperly [1]. Even a normal ECG and negative enzymes in a low risk patient with history of recent chest pain need to be reassessed repeatedly, as stated in recent guidelines [2], searching for suggestive ECG alteration even during asymptomatic period.

## 2. Case Report

**Figure 1:**
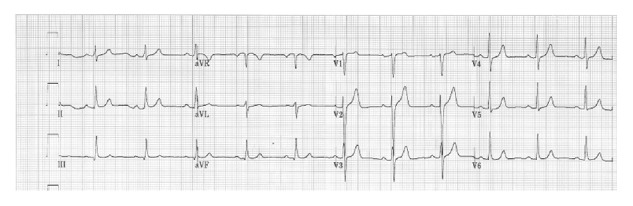
ECG during chest pain.

In May 2014, a 48-year-old male, working as bus driver, underwent occupational evaluation. He was asymptomatic for chest pain and dyspnea and declared smoking habits and no history of other cardiovascular risk factors. He was HIV positive. He referred to a previous access for a twenty-minute-long chest pain episode at emergency department two months before; nevertheless he was discharged after six hours on the basis of a normal ECG (Figure 1) and negative serum cardiac markers, with indication for ambulatory exercise stress test and invitation to stop smoking. Yet in the subsequent period he felt well and decided to hold over any other examination.

**Figure 2:**
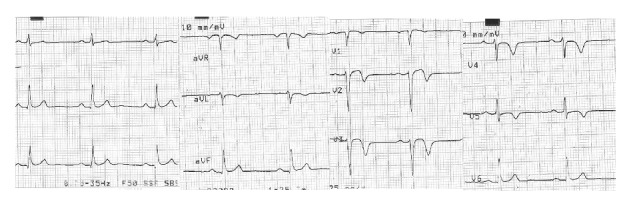
ECG during asymptomatic period.

During the occupational evaluation the routine ECG showed sinus rhythm, 60 bpm, and normal R-wave progression in peripheral and precordial leads, with biphasic T wave in V2-V3 and deeply inverted T wave in V4-V5; negative T wave was also present in aVL (Figure 2). Therefore he was advised with early cardiological assessment. These findings were suggestive of Wellens ECG pattern [3], strongly associated with critical stenosis of proximal left anterior descending artery, meaning a very large amount of myocardial tissue at risk of necrosis and subsequent sudden death.

**Figure 3:**
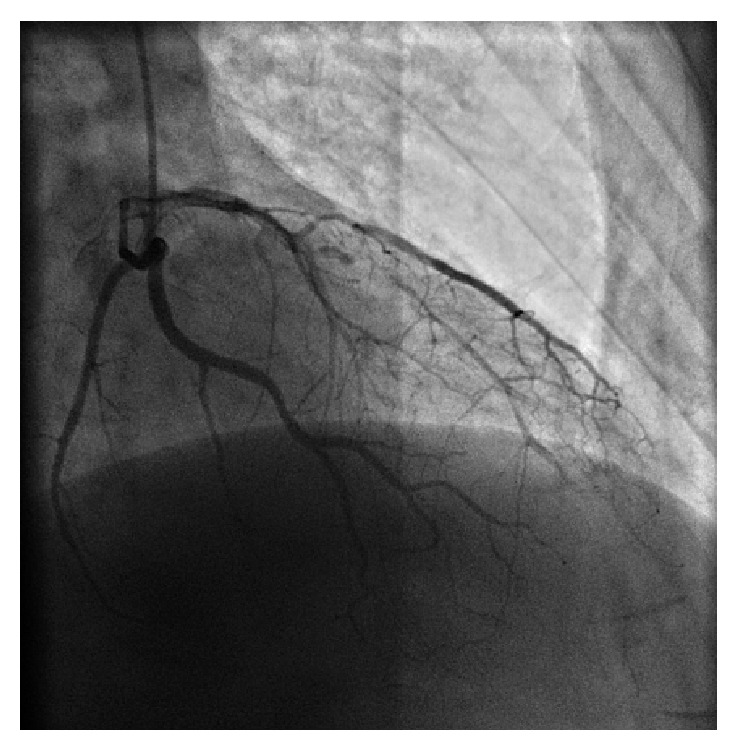
Coronary angiography.

**Figure 4:**
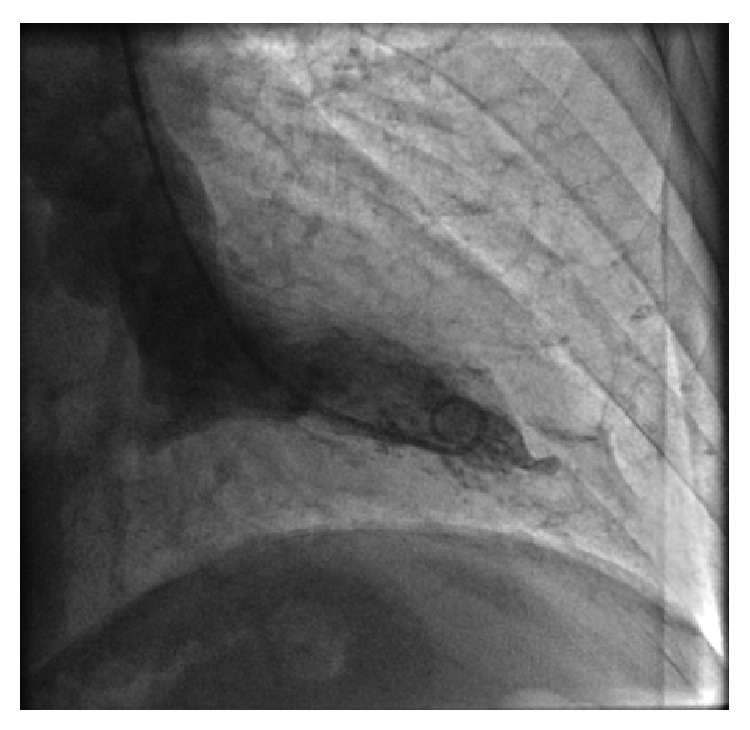
Cardiac ventriculography.

In this case a rest echocardiography showed no regional contractility anomaly without evidence of valvular and pericardial disease. Thus the patient was admitted to cardiovascular department with indication for coronary angiography. The exam revealed critical stenosis of proximal left anterior descending (LAD) artery, first and second diagonal branch, and subcritical stenosis of intermediate branch (Figure 3); at same time ventriculography confirmed normal ventricular contractility (Figure 4). He underwent successful percutaneous coronary angioplasty with placement of multiple drug eluting stent at critical lesions levels and discharged safely two days after with indication of best drug therapy, strong advice in smoke cessation, and regular cardiovascular follow-up.

Wellens' syndrome is characterized by previous history of angina, minimal or no elevation of serum cardiac markers, biphasic or deeply inverted T wave in leads V2-V3, sometimes extended in leads V1 and V4–V6, no pathological Q wave, no loss of precordial R-wave progression, and minimal or no ST-segment elevation during pain-free presentation and it is recurrently associated with pseudonormalization of the described pattern during angina. As underlined by Movahed [4], this electrocardiographic cluster, associated with critical LAD stenosis, was first described in patients undergoing exercise stress test by Gerston in 1979, who named it “exercise induced U-wave inversion,” although the ECG alterations only interest the latter phase of T wave; Wellens [3] described the same pattern at rest two years later in a more extensive population affected by angina, obtaining the world wide eponym for the syndrome, still described in both terminologies by different authors. The pathophysiology of this ECG genesis is likely due to repetitive and uneventful episode of transmural myocardial ischemia-reperfusion in the large territory of left anterior descending artery, associated with edema of myocardial wall [5], while the described T wave pseudonormalization during angina is the expression of the ongoing large transmural myocardial ischemia [6]. The prompt recognition of this ECG pattern has paramount implication for the correct management of the patient, as it likely underlines the presence of proximal LAD critical stenosis [7], avoiding both acute complication and chronic coronary disease progression. This consideration implies both early coronary angiography in pain-free patient presenting at outpatient clinic with this ECG characteristics (after careful exclusion of other myocardial and pericardial cause by echocardiography) and prolonged and mindful observation of ECG pattern variation in chest pain patient with normal ECG presenting at emergency department in the close period following angina. An inappropriate discharge in this setting will easily translate into a very high risk of myocardial infarction and sudden death; at same time the indication for exercise test would represent the double hazard of delayed diagnosis and lethal ischemia-related ventricular arrhythmias.

## 3. Conclusion

We describe a lucky case of Wellens' syndrome correctly recognized only two months after early presentation at emergency department. We underline, according to current guidelines, the careful and prolonged patient observation in chest pain unit after a symptomatic episode and repetitive ECG evaluation also during pain-free period to exclude a T wave pseudonormalization during angina. The quick recognition of this particular ECG pattern will translate into a prompt assessment for coronary artery disease and into a better prognosis for our patients. Furthermore, we believe that a well organized outpatient clinical pathway after discharge would avoid a follow-up drop out of these patients, related to social and cultural environment.
